# A chromosome-level genome assembly of tomato pinworm, Tuta absoluta

**DOI:** 10.1038/s41597-023-02299-5

**Published:** 2023-06-17

**Authors:** Ying Liu, Xi Chen, Yanqiong Yin, Xiaowei Li, Kang He, Xueqing Zhao, Xiangyong Li, Xiyan Luo, Yang Mei, Zuoqi Wang, Runguo Shu, Ziqi Cheng, Kifle Gebreegziabiher Gebretsadik, Chen Luo, Ran Wang, Yaobin Lv, Aidong Chen, Fei Li

**Affiliations:** 1 Key Laboratory of Green Prevention and Control of Agricultural Transboundary Pests of Yunnan Province and Agricultural Environment/ Agriculture Environment and Resources Institute, Yunnan Academy of Agricultural Sciences, Kunming, 650205 China; 2 State Key Laboratory of Rice Biology & Ministry of Agricultural and Rural Affairs Key Laboratory of Molecular Biology of Crop Pathogens and Insects & Key Laboratory of Biology of Crop Pathogens and Insects of Zhejiang Province, Institute of Insect Sciences, Zhejiang University, Hangzhou, 310058 China; 3 Institute of Plant Protection and Microbiology, Zhejiang Academy of Agricultural Sciences, Hangzhou, 310021 China; 4 Tigray Agricultural Research Institute (TARI), Mek’ele, Tigray +492 Ethiopia; 5 Institute of Plant Protection, Beijing Academy of Agriculture and Forestry Sciences, Beijing, 100097 China

**Keywords:** Entomology, Agricultural genetics

## Abstract

The tomato pinworm, Tuta absoluta, or Phthorimaea absouta, is native to South America, but quickly spread to other regions of world, including Europe, Africa, and Asia, devastating to global tomato production. However, a lack of high-quality genome resources makes it difficult to understand its high invasiveness and ecological adaptation. Here, we sequenced the genome of the tomato pinworm using Nanopore platforms, yielding a genome assembly of 564.5 Mb with contig N50 of 3.33 Mb. BUSCO analysis demonstrated that this genome assembly has a high-level completeness of 98.0% gene coverage. In total, 310 Mb are repeating sequences accounting for 54.8% of genome assembly, and 21,979 protein-coding genes are annotated. Next, we used the Hi-C technique to anchor 295 contigs to 29 chromosomes, yielding a chromosome-level genome assembly with a scaffold N50 of 20.7 Mb. In sum, the high-quality genome assembly of the tomato pinworm is a useful gene resource that contributes to a better understanding of the biological characteristics of its invasiveness and will help in developing an efficient control policy.

## Background & Summary

The tomato pinworm, *Tuta absoluta*, also called *Phthorimaea absouta*, is a moth in the family Gelechiidae and is a destructive pest on tomato crops^1^. It originated in South America and was detected in eastern Spain toward the end of 2006^2–4^. Since then, it has rapidly spread to Europe and North Africa, where it has become a notorious pest threatening tomato production in both greenhouses and outdoors. It was first detected in Turkey in 2009, and has successfully spread to Asia^5^. It then spread to Europe and Asian countries, such as Russia, Kazakhstan, Kyrgyzstan, Tajikistan, Pakistan, India, Nepal, and Bangladesh^6–8^. In 2017, the first case of *T. absoluta* in China was detected in the Xinjiang Uygur Autonomous Region which is the largest tomato production region of China^9^. In early 2018, it caused significant damage in the Lincang region of Yunnan province, another tomato main production area in China^9^. *T. absoluta* has now been found in over 90 countries and has become a serious threat to global tomato production.

*T. absoluta* is harmful at any development stage and afflicts almost every part of the tomato plant. It feeds on the mesophyll by diving into the leaves, mainly through larvae, and forms a dive path on the leaves^4^. It negatively affects plant photosynthesis, and in serious cases, can lead to plant leaves shrinking, drying, and falling off. When larvae attack the young stem of the plant, the plant cracks and seriously affects overall tomato production. The larvae penetrate the inside of the tomato fruit and cause harm, forming feeding spots on the surface or causing the fruit to become smaller and deformed, making it less economically valuable^10^. It has been estimated that *T. absoluta* could cause an 80% to 100% loss of tomato yield if no pest control action is taken^3^. Therefore, the rapid expansion of *T. absoluta* has resulted in yield losses, fruit quality reduction, increases in the cost of pest control, and the overuse of chemical insecticides^4,8,11^. Moreover, *T. absoluta* can also damage other solanaceous species, such as eggplant, pepper and tobacco^6,12,13^.

Chemical control is the main management strategy against *T. absoluta*^8,14^. Particularly, in newly invaded areas, large quantities of pesticides are used to control *T. absoluta* to reduce yield loss^4,15^. Unfortunately, the heavy use of insecticides has reduced the field population of naturally beneficial arthropods^16^, and led to the rapid development of insecticide resistance in *T. absoluta* populations^17–20^. Genome analysis has been proven to be helpful for developing pest control strategies to control *agricultural pests*^21,22^. However, the reported *T. absoluta* genome is not of high quality^23^, which hinders the full use of genome resources. Here, we generated a chromosome-level genome assembly of *T. absoluta* using Nanopore sequencing and Hi-C technology. Tomato pinworm genomes show high chromosomal synteny with silkworms and fall armyworms. The widespread ecological adaptation of the tomato pinworm can be partially explained by the expansion of gene families associated with detoxification metabolism^24^. The high-quality genome assembly (assessed by 3 C criterion)^25^ of the tomato pinworm provides a useful data resource for the in-depth analysis of insect invasion, chromosome rearrangement, evolution, and pest control.

**Fig. 1:**
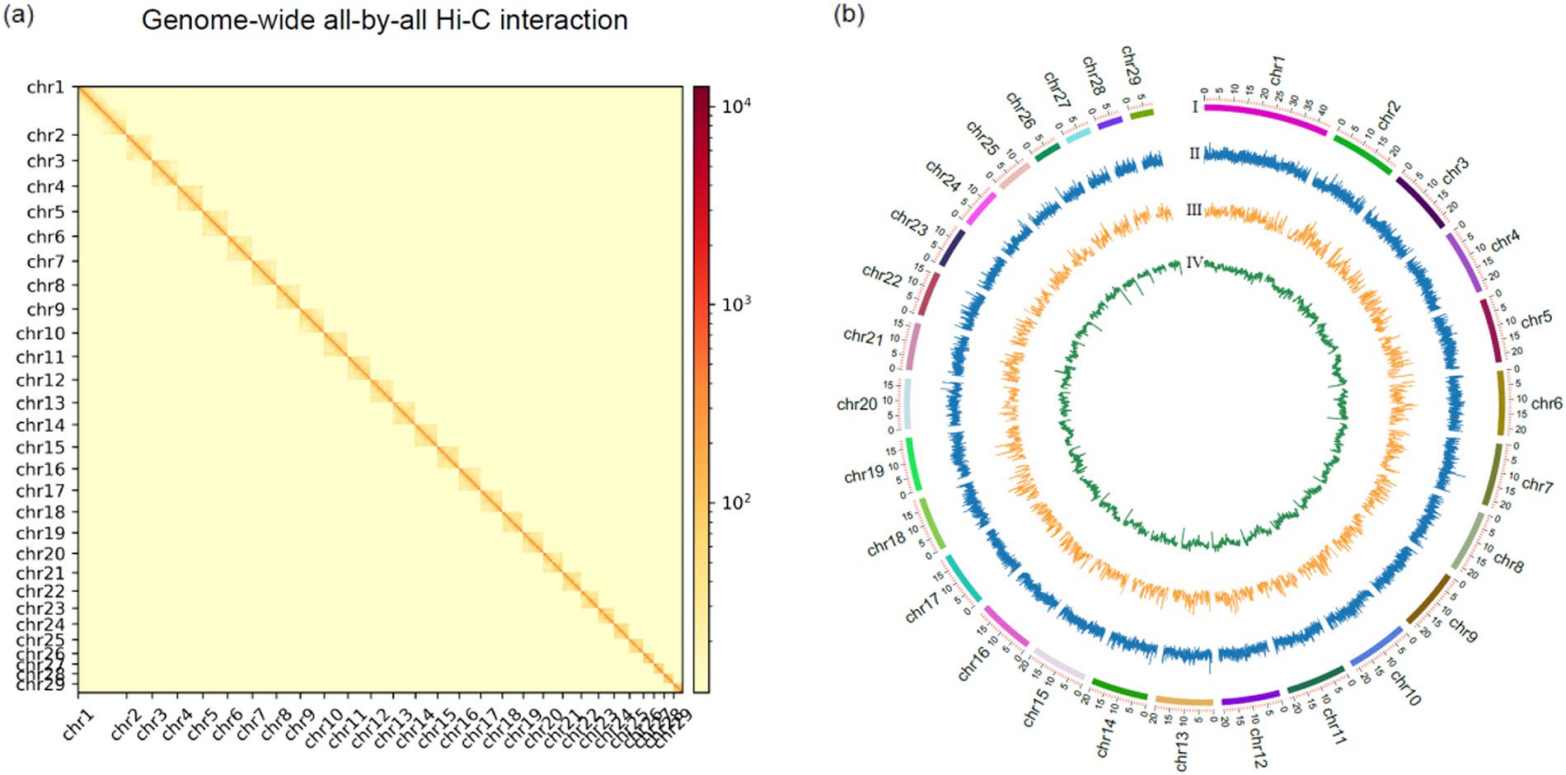
Heatmap of genome-wide Hi-C data and overview of the genomic landscape of the South American tomato pinworm, *T. absoluta*. (**a**) The heatmap shows all interactions between 29 chromosomes of the South American tomato pinworm. There were strong intra-chromosomal interactions (blocks on the diagonal line), while inter-chromosomal interactions were weaker. The frequency of Hi-C interaction links is represented by the color, which ranges from yellow (low) to red (high). (**b**) Blocks on the outmost circle represent all 29 chromosomes of the South American tomato pinworm. Peak plots from outer to inner circles in blue, yellow, and green represent GC content, gene density, and a repeat sequence coverage of each chromosome, respectively (Sliding window size = 200 Kb).

The self-corrected and polished Nanopore reads were used to assemble a draft genome assembly with a total length of 564.5 Mb, consisting of 301 Contigs with an N50 length of 3.3 Mb. The assembled genome size is generally consistent with that estimated by flow cytometry (581.9 Mb) (Fig. S1). To evaluate the quality of the genome assembly, a total of 98.14% of the short reads were uniquely mapped to the genome assembly and the coverage rate was 99.7%, indicating that the assembled genome was of high quality (Table 1). These evaluations indicated that the genome assembly had a high level of completeness and was suitable for subsequent analysis. Next, we used Hi-C sequencing to orientate and anchor Contigs to scaffolds. After HIC assembly and manual curation, 98.14% of the total sequence length from the genome assembly has been successfully assigned to the 29 chromosomes (Fig. 1a). The longest chromosome, chromosome 1, has a length of 44.3 Mb, while the shortest chromosome, chromosome 29, has a length of 8.2 Mb. The remaining part represents scaffolds that have not been assigned to any specific chromosome location. The chromosome-level genome assembly was 564.5 Mb with a scaffold N50 of 20.7 Mb. (Chromosomal-level assembly means that it includes the assembly of the 29 chromosomes. However, it’s important to note that the remaining scaffolds, which were not specifically localized to the 29 chromosomes, also contain valuable information and are included in our chromosomal-level assembly. They have not been excluded.) BUSCO v3.0.2b was used to estimate the completeness and contiguity of the genome assembly. The insect_db9 dataset was selected as the library. The results demonstrated that 98.0% of BUSCO genes could be successfully detected, of which 97.3% are single-copied and 0.7% are duplicated (Table 2). Quality evaluations indicated that the genome assembly had high completeness and was suitable for subsequent analysis.

**Table 1:** Assessment of correctness of the *T. absoluta* assembly (Mapping Rate and Coverage of Short-read Sequencing Data).

Type	Percent
Map rate	98.14%
Average depth	174.362
Coverage	99.721%

**Table 2:** Assessment of completeness of the *T. absoluta* assembly.

Category	Number of BUSCOs
C: 98.0% [S: 97.3%, D: 0.7%], F: 0.4%, M: 1.6%	5,286
Complete BUSCOs (C)	5,181
Complete and single‐copy BUSCOs (S)	5,142
Complete and duplicated BUSCOs (D)	39
Fragmented BUSCOs (F)	21
Missing BUSCOs (M)	84

**Fig. 2:**
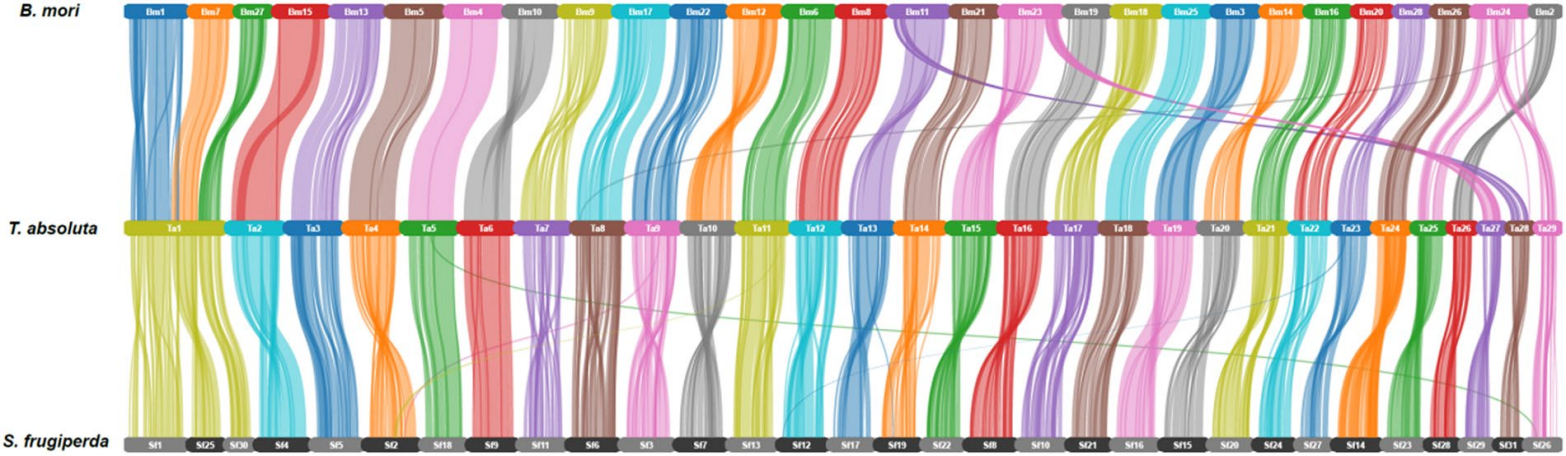
Chromosome-level synteny analysis. Chromosome-level synteny analysis of the South American tomato pinworm (*T. absoluta*) and two lepidopteran insects: silkworm (*B. mori*) and fall armyworm (*S*. *frugiperda*).

We compared the syntenic relationships between the South American tomato pinworm (*T. absoluta*) and two other lepidopterans, including the silkworm (*Bombyx mori*), and fall armyworm (*Spodoptera frugiperda*). Though these lepidopteran insects generally shared high chromosomal synteny, we detected several fusion and fission events between *T. absoluta* and the other lepidopteran insects (Fig. 2). The South American tomato pinworm chromosome 1 is syntenic to a large portion of the Z chromosome (Chr1) together with Chr7 and Chr27 of the silkworm; a large portion of the Z chromosome (Chr1) and a small fragment of Chr25 and Chr30 of the fall armyworm.

The repeat sequences were annotated. In total, 54.8% of the South American tomato pinworm genome was annotated as repeat sequences (Fig. 1b). Short interspersed nuclear elements (SINEs), long interspersed nuclear elements (LINEs), long terminal repeats (LTRs), and DNA transposons accounted for 8.61%, 13.25%, 4.84%, and 4.05% of the whole genome, respectively, and 16.95% of repeat sequences were annotated as unclassified. After masking repeat sequences, a total of 21,979 protein-coding genes were annotated (Table 3). Of all predicted genes, 14,877 had annotation information. Furthermore, 8,769 genes were assigned with GO terms and 8,373 genes were mapped to at least one KEGG pathway.

**Table 3:** Assessment of contiguity of genome assemblies of *T. absoluta* and other five lepidopteran insects.

Species	*T. absoluta*	*B. mori*^39^	*C. pomonella*^21^	*C. suppressalis*^69^	*D. plexippus*^70^	*S. exigua*^57^
Assembly size (Mb)	564.5	460.3	772.9	825.7	248.7	446.8
Karyotype	N = 30	N = 28	N = 28	N = 29	N = 30	N = 31
Number of assembled chromosomes	28 A + Z	26 A + Z	27 A + Z + W	28 A + Z	29 A + Z	31 A + Z + W
Contig N50 size (Mb)	3.3	12.2	0.8	0.3	0.1	—
Scaffold N50 size (Mb)	20.7	16.8	8.9	27.1	9.2	14.4
Protein‐coding genes	21,979	16,880	17,184	15,653	19,762	17,707
Repeats (%)	54.8	46.8	42.9	46.4	—	33.1
GC (%)	38.5	38.3	37.4	34.9	32.1	36.7

**Fig. 3:**
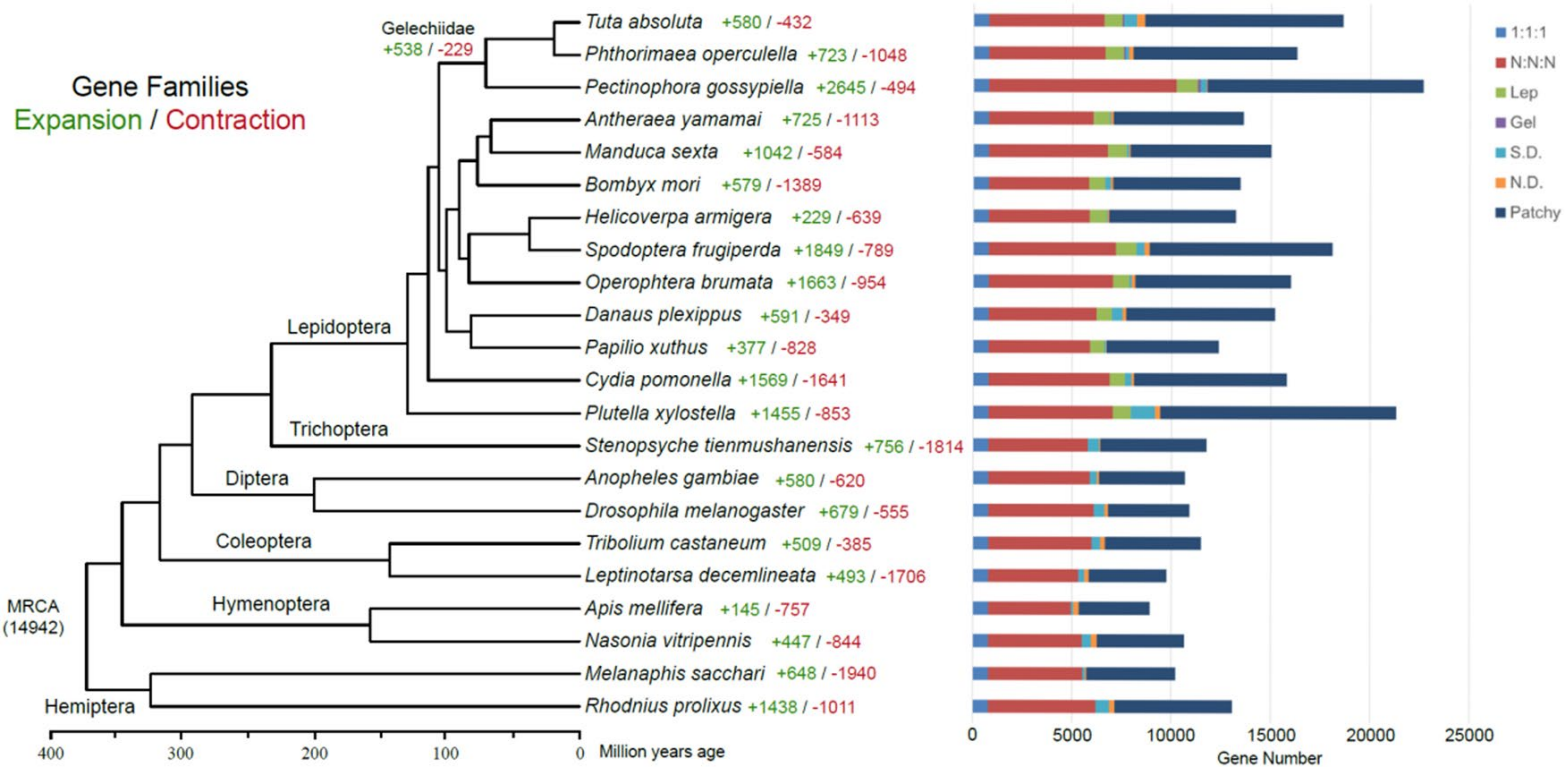
Phylogenetic tree and gene orthology. A phylogenetic tree of the South American tomato pinworm *T. absoluta* and other insect species was constructed using the maximum likelihood method with concatenated protein sequences of 229 single-copy orthologous genes with 1,000 bootstrap replicates. The numbers of expanded TreeFam gene families (green) and contracted TreeFam gene families (red) are shown to the right of each species branch. MRCA is the most recent common ancestor. The colored bars to the right represent the number of genes classified into seven orthology types. “1:1:1” represents universal single-copy genes in all species, with absence and/or duplication in no more than one genome; “N:N:N” represents other universal genes; “Lep.” represents unique genes common to Lepidoptera; “Gel.” represents unique genes common to the Gelechiidae family; “S.D.” represents species-specific duplication; “N.D.” represents species-specific genes; “Patchy” represents all other genes.

An orthologous group (orthogroup) is the set of genes derived from a single gene in the last common ancestor of all the species under consideration. A total of 15,027 orthologous groups with 229 single-copy orthologous groups were identified among *T. absoluta* and other 21 insect species by orthofinder; the number of genes assigned to different orthologous groups is displayed in Fig. 3. A phylogenetic tree generated using protein-coding sequences of single-copy orthologous genes showed that the tomato pinworm and thirteen other moths were clustered together (Fig. 3). The tomato pinworm (*T. absoluta*), the potato tuber moth (*P.operculella*) and the pink bollworm (*P. gossypiella*) are members of the Gelechiidae family. The split of the Gelechiidae lineage from other lepidopteran clusters was inferred to be around 122.9 million years ago (Mya). All 13 lepidopteran insects diverged from the sister lineage caddisfly (*S. tienmushanensis*) approximately 267.3 Mya ago (Fig. 3), which is consistent with a previous report^26^.

We used CAFE software to study the expansion and contraction of TreeFam gene families (Fig. 3). Of the 6,464 gene families in the Most Recent Common Ancestor (MRCA) of all 22 species, 580 were expanded and 432 were contracted in the tomato pinworm compared with their common ancestor. It has lower levels of expansion and contraction compared with other members of Lepidoptera, which suggests that it is not a result of fast and intense evolutionary events. GO enrichment analysis of the 580 expanded TreeFam families in tomato pinworms showed that these genes were enriched in processes including vesicle-mediated intercellular transport (GO: 0110077, 10 genes, p = 2.58E-22, FDR-adjust), intercellular transport (GO: 0010496, 10 genes, p = 1.01E-18, FDR-adjust), response to starvation (GO: 0042594, 15 genes, p = 4.59E-11, FDR-adjust) and regulation of neuronal synaptic plasticity (GO: 0048168, 23 genes, p = 4.59E-11, FDR-adjust) (Table S2). GO analysis demonstrated that the 432 contracted TreeFam gene families were significantly enriched in the estrogen catabolic process (GO: 0006711, 11 genes, p = 2.21E-18, FDR-adjust), bilirubin conjugation (GO: 0006711, 11 genes, p = 1.95E-19, FDR-adjust), and lipid catabolic process (GO: 0016042, 15 genes, 1.61E-17, FDR-adjust) (Table S3). However, further investigations are still needed to determine the functions associated with the genes in these expanded and contracted gene families, such as analysis of their expression patterns and their putative roles in ecological adaptation-associated processes such as invasion.

**Fig. 4:**
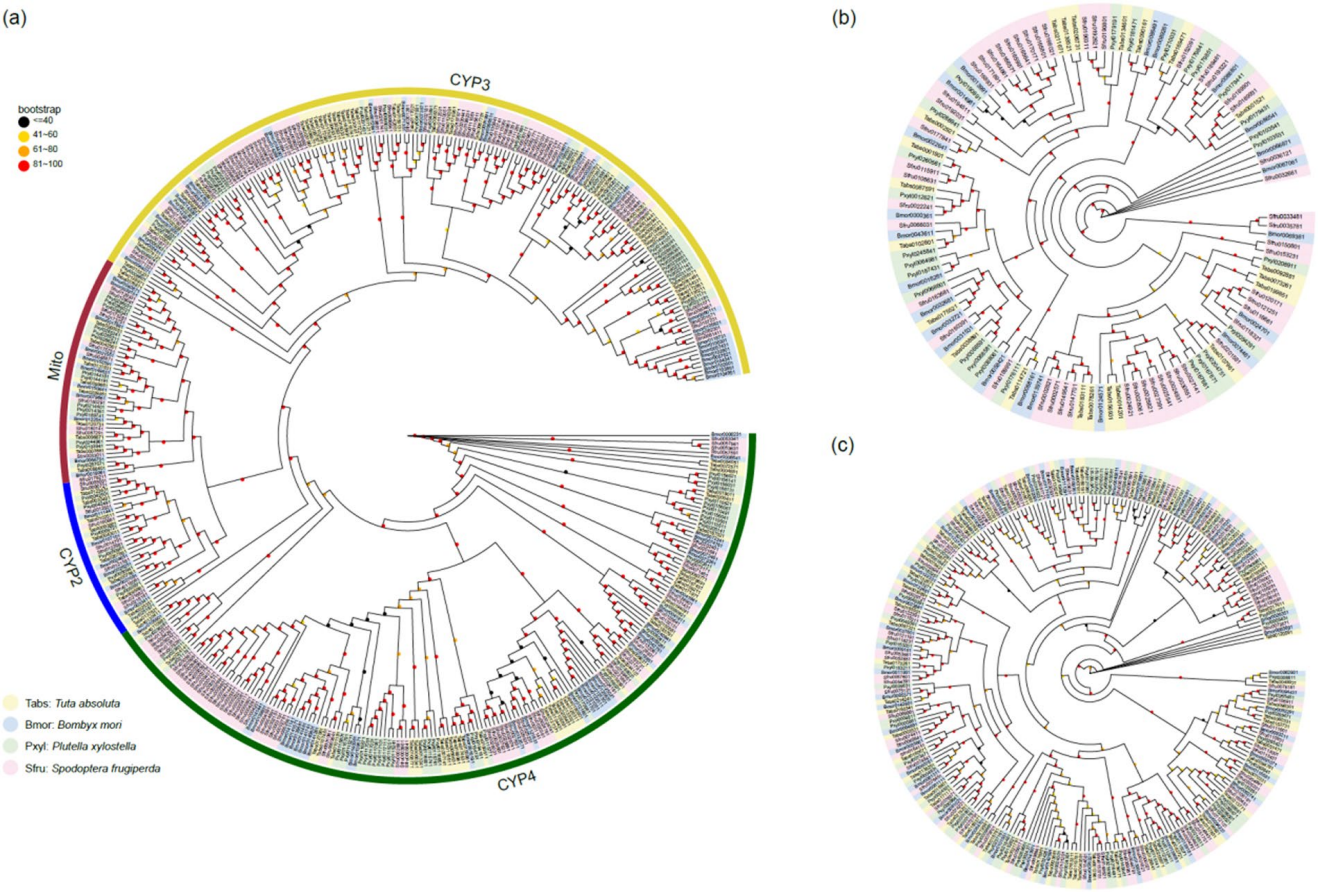
Detoxification-related gene families in four Lepidopteran species. (**a**) Maximum-likelihood phylogenetic analysis of cytochrome P450 gene family. (**b**) Maximum-likelihood phylogenetic analysis of glutathione *S*-transferases gene family. (**c**) Maximum-likelihood phylogenetic analysis of ATP-binding cassette transporters gene family.

The expansion of the cytochrome P450 gene family is a main contributor to rapid adaptation of insects^24^. With this chromosome-level genome, we identified 104 cytochrome P450 genes in tomato pinworms by TBLASTN and Genewise, which is greater than most other insects. Phylogenetic analysis indicated that P450 clan 3 shows an expansion in tomato pinworm compared with silkworm (Fig. 4a), while P450 clans Mito and 2 were strongly conserved in Lepidopteran insects. Based on the previous analysis, *T. absoluta* was likely to experience rapid adaptation and evolution in detoxification. Other detoxification-related gene families like glutathione S-transferases (GSTs) and ATP-binding cassette transporters (ABC transporters) do not show any sign of expansion or contraction (Fig. 4b,c).

## Methods

### Sample collection

*T. absoluta* pupae were collected from a tomato field in Midu, Yunnan Province in July 2021, and were maintained at the Yunnan Academy of Agricultural Sciences. The insects were fed with fresh tomato seedlings and maintained at 26 ± 1 °C, a 14:10 (L:D) photoperiod cycle, and 85% ± 5% relative humidity. Five generations were reared and the pupae of the fifth generation were used for sequencing.

### Genome size estimation

The genome size was evaluated by flow cytometry using He’s method^27^. The heads of five female adults were dissected for the experiment. The fruit fly Drosophila melanogaster Canton-S strain was used as the reference species with a standard genome size of 1 C = 176.4 Mb. First, we prepared the Galbraith buffer which releases the nuclei of cells, including 45 mM MgCl2, 30 mM sodium citrate, 20 mM 3-[N-morpholino] propane sulfonic acid (MOPS) and 1 L ddH2O with 1 ml Triton X-100. The Galbraith buffer was adjusted to pH 7.0 using HCl and then filtered through a 0.22-μm nylon filter before it was used. Next, we put the fly or *T. absoluta* heads into 500 μl Galbraith Buffer and chopped the tissues to obtain the mixed solution, which was filtered through 40U nylon mesh to remove the impurities. The filtrate was then centrifuged at 2,000 g and 4 °C for 10 min. Then, we removed the supernatant and added 500 μl Phosphate Buffered Saline (PBS) to the tubes, shook them and added 10 μg RNase A into each tube. After 10 minutes, we stained the solution of each tube with 25 μg propidium iodide and covered them with tin foil paper. Then, the solution was placed in the dark for at least 15 minutes. The flow cytometry was conducted on a Partec Cyflow cytometer. All experiments were repeated in triplicate.

### Genome sequencing and assembly

Female pupae were collected and rinsed with pre-cooled 0.9% saline to avoid contamination before being frozen with liquid nitrogen. A total of 10.3 µg genomic DNA was extracted from a female pupa using the sodium dodecyl sulfate (SDS) extraction method^28^. After the DNA quality and integrity was tested, it was randomly sheared by Covaris ultrasonic disruptor. Illumina sequencing pair-end libraries with insert size of 300 bp were prepared using Nextera DNA Flex Library Prep Kit (Illumina, San Diego, CA, USA). Sequencing was performed using the Illumina NovaSeq platform (Illumina, San Diego, CA, USA). We filtered the raw reads using fastp software (version 0.21.0) with the following criteria: Removal of reads with an N base content exceeding 5%; Discarding reads with a low-quality base count of 50% or more, where the quality value is less than or equal to 5; Removal of reads containing adapter contamination; Elimination of duplicate sequences caused by PCR amplification. For Oxford Nanopore sequencing, the libraries were prepared using the SQK-LSK109 ligation kit and using the standard protocol. The purified library was loaded onto primed R9.4 Spot-On Flow Cells and sequenced using a PromethION sequencer (Oxford Nanopore Technologies, Oxford, UK) with 48-h runs at Wuhan Benagen Technology Co., Ltd., Wuhan, China. We then performed quality assessment of the raw data using Oxford Nanopore GUPPY software (version 0.3.0) (https://timkahlke.github.io/LongRead_tutorials/BS_G.html) and filtered out low-quality reads with sequencing quality value (Q) less than 7, resulting in the retention of high-quality pass reads.

The draft genome was assembled using the raw reads of the Nanopore and Illumina sequencing platform. First, we used the NextDenovo software (https://github.com/nextomics/nextdenovo) and the error corrected long reads to produce a draft genome assembly. Next, we used ONT sequencing data to perform two rounds of self-error correction of the draft assembly with the software Racon v1.4.11^29^. Lastly, second-generation sequencing data were used to perform two rounds of error correction for the draft genome assembly from the third-generation long reads with self-correction. The software Pilon v1.23 was used with default parameters^30^. For assessment of correctness, the clean Illumina short reads were mapped to the assembly profile using BWA v0.6.2^31^. Assessment of assembly completeness was generated using BUSCO v3.0.2b^32^.

### Hi-C scaffolding

Using one female pupa as the input, Hi-C libraries were constructed following previously described standard protocols^33^. To optimize permeation, the sample was cut into pieces. Tissues were ground with liquid nitrogen and then incubated for 30 minutes in a 4% formaldehyde solution at room temperature in a vacuum. We quenched the crosslinking reaction for 5 minutes with glycine (2.5 M), then placed samples on ice for 15 minutes. Following centrifugation at 2,500 rpm for 10 minutes at 4 °C, the pellet was washed with 500 µl PBS and centrifuged for 5 minutes at 2,500 rpm. After resuspending the pellet in 20 ul of lysis buffer, the supernatant was centrifuged at 5000 rpm for 10 minutes at room temperature. We washed the pellet twice in 100 µl ice-cold 1x NEB buffer and centrifuged it for 5 minutes at 5,000 rpm. The nuclei were re-suspended in 100 µl NEB buffer, solubilized with dilute SDS, and incubated at 65 °C for 10 min. The samples were digested overnight at 37 °C on a rocking platform with a 4-cutter restriction enzyme, MboI (400 units), after quenching the SDS with Triton X-100.

DNA ends were marked with biotin-14-dCTP and blunt-end ligation was used. A ligation enzyme was added to re-ligate the proximal chromatin DNA. Proteinase K was used to reverse cross-link the nuclear complexes at 65 °C. DNA was purified by phenol-chloroform extraction. T4 DNA polymerase was used to remove biotin from non-ligated fragment ends. The ends of fragments sheared by sonication (200–600 base pairs) were repaired with a mixture of T4 DNA polymerase, T4 polynucleotide kinase and Klenow DNA polymerase. Streptavidin C1 magnetic beads were used to enrich biotin-labeled Hi-C samples. Ligation of Illumina paired-end sequencing adapters follows the addition of A-tails to fragment ends. The Hi-C sequencing library was amplified by PCR (12–14 cycles) and sequenced on the Illumina NovaSeq platform after quality control.

The high-quality sequencing reads were mapped to the draft genome by BWA v0.6.2^31^. Unmapped paired-end reads and multiple mapped reads were filtered by Samtools v1.9^34^. The unique high-quality paired-end reads mapped close to the restriction sites were retained for downstream analysis. ALLHIC^35^, 3D-DNA^36^, juicer^37^, and Juicebox^38^ were used to cluster the scaffolds into groups, and the order of the scaffolds was confirmed by the strength of interactions between read pairs and was checked and corrected manually. Orientations were assigned to each group.

### Synteny analysis

The genome data of silkworm *Bombyx mori*^39^, and fall armyworm *Spodoptera frugiperda*^40^ were obtained from InsectBase 2.0^41^. For synteny analysis, we performed a BLAST search of annotated protein sequences using DIAMOND v2.2.22^42^ with default parameters. MCScanX^43^ with the parameters “-s10 -b 2.” was used to identify synteny information. The results were visualized with SynVisio (https://synvisio.github.io/#/).

### Genome annotation

For repeat sequence annotation, we first constructed a de novo repeat library using RepeatModeler v2.0.2a with the LTR structural discovery pipeline^44^. We then masked repeat sequences across the *T. absoluta* genome using RepeatMasker v4.1.2^45^ against both the de novo species-specific repeat library generated by RepeatModeler2 and the RepBase v26.03 library^46^. After masking these repeat sequences, ab initio prediction, homology searching and transcriptome-based approaches were integrated to predict protein-coding genes. For transcriptome-based prediction, HISAT v2.1^47^ was used to align the transcriptome data to the genome, and gene information was predicted using StringTie v1.3.4c^48^. For the homology-based approaches, the annotated gene sets from all invertebrate species (downloaded from the National Center for Biotechnology Information [NCBI] Refseq database) were downloaded. Then the protein-coding genes were annotated by the BRAKER2^49^ pipeline D using de novo, homology-based protein evidence, and RNA-Seq alignment information. We used eggNOG-mapper v2^50^ to perform functional annotation and clusterProfiler 4.0^51^ to perform enrichment analysis. We also searched the SwissProt and NCBI non-redundant databases using DIAMOND v2.2.22 (E-value < 1E-5)^42^.

### Comparative genomics and phylogenetic reconstruction

We downloaded all of the protein-coding gene sequences of 22 insects from InsectBase to perform phylogenomic analysis, covering six insect orders, Lepidoptera, Trichoptera, Diptera, Coleoptera, Hymenoptera, and Hemiptera. The protein sequences were used for phylogenomic analysis (Table S1), which were all collected from InsectBase 2.0. We only kept the longest transcript of each gene for analysis. OrthoFinder v2.3.14^52^ was used with DIAMOND v2.2.22^42^ with default settings to identify orthologs and homologs.

To infer the phylogeny of these insects, multiple sequence alignments of single-copy gene families were performed using MAFFT v7.310^53^ with the “-auto” parameter, and trimming was performed by trimAL 1.2^54^ with the “-automated1” setting. The alignment results were concatenated to construct a maximum likelihood phylogenetic tree using IQTREE v2.2.0^55^ with Q.insect model. Statistical support was obtained with 1000 bootstrap replicates. Divergence information was taken from the TimeTree^56^ database (*Rhodnius prolixus* vs *B. mori* 330–481 Mya, *Phthorimaea operculella* vs *B. mori* 91–103 Mya, *Manduca sexta* vs *Tribolium castaneum* 281–361 Mya, *Apis mellifera* vs *Pectinophora gossypiella* 313–390 Mya and *Drosophila melanogaster* vs *B. mori* 224–345 Mya) and this was then used to constrain the divergence estimate with R8s v1.81^57^. The tree was visualized using Evolview 2.0^58^.

### Gene family expansion and contraction

The TreeFam database was used to analyze the gene number of each gene family in each species^59,60^. The resulting matrix tables were used as inputs to examine the expansion and contraction of each gene family in CAFE v4.2.1 with a p-value < 0.05 as the cut-off^61^.

### Gene family analysis

For the P450 gene family, the reference protein sequences of lepidoptera P450s were downloaded from InsectBase 2.0 and manually confirmed to construct a clean and reliable P450 dataset. Then, we used TBLASTN (blast v2.12.0) to search candidate P450s in the fall armyworm genome assembly (E-value < 1E-5). Genewise v2.4.1 was used to identify the gene structure^62^. Additionally, these candidate sequences were confirmed by HMMER v3.2.1^63^ to verify their P450 domain (Pfam domain PF00067, E-value < 1E-5) (Bateman, *et al*. 2004). To classify these P450 sequences into specific groups (CYP2, CYP3, CYP4, Mito), we compared the South American tomato pinworm P450 sequences to the P450 genes of *B. mori*, *S. frugiperda* and *P. xylostella* using NCBI-BLAST (E-value < 1E−5).

For other gene families including glutathione *S*-transferases (GSTs) and ATP-binding cassette transporters (ABC transporters), we identified each gene family’s genes using a two-step method in OGS. First, we collected the reference protein sequences of each gene family from InsectBase 2.0 and NCBI GenBank, which were further manually confirmed. Then, we used BLASTP to obtain candidate sequences from the OGS of each insect (E-value < 1E-5). Next, HMMER was used to align the candidate sequences to the Pfam database (E-value < 1E-5)^64^.

For the phylogenetic analysis of gene families, we aligned protein sequences of each gene family using MAFFT v7 and filtered sequences with trimAl v1.2 to obtain the conserved domains. IQ-Tree was used to construct the phylogenetic tree with the best model (LG + R8) estimated by ModelFinder (1000 ultrafast bootstrap approximation replicates)^65^. The tree was visualized using Evolview 2.0^58^.

## Data Records

The Nanopore, Hi-C and Illunina sequencing data that were used for the genome assembly and annotation have been deposited in the NCBI Sequence Read Archive with accession number SRP418788^66^. The final chromosome assembly has been deposited at GenBank under the accession GCA_029230345.1^67^. The final chromosome assembly and OGSv1 were submitted to InsectBase 2.0 (http://v2.insect-genome.com/Tabs)^68^.

## Technical Validation

We use the 3 C (Contiguity, Completeness, and Correctness) criterion to comprehensively assess the quality of our genome assembly^25^. The chromosome-level genome assembly was 564.5 Mb with a scaffold N50 of 20.7 Mb. For quantitative assessment of genome assembly, BUSCO assessment showed that 98% of BUSCO genes (insecta_db9) were successfully identified in the genome assembly (Table 2), suggesting a remarkably complete assembly of the *T. absoluta* genome.

The Hi-C heatmap revealed a well-organized interaction contact pattern along the diagonals within/around the chromosome inversion region (Fig. 1), which indirectly confirmed the accuracy of the chromosome assembly.

## Supplementary information

Supplement_file

## Data Availability

All software and pipelines were executed according to the manual and protocols of the published bioinformatic tools. The version and code/parameters of software have been described in Methods.
