# Supplementary material for: A chromosome-level genome assembly of tomato pinworm, Tuta absoluta
Source: Sci Data. 2023 Jun 17;10:390. doi: 10.1038/s41597-023-02299-5 (PMC10276875; doi:10.1038/s41597-023-02299-5)
Supplement: Supplementary file 1 — Supplement_file [file 41597_2023_2299_MOESM1_ESM.docx]

**Contents**

| **Title** | **Page** |
| --- | --- |
| Figure S1 | 2 |
| Table S1 | 3 |
| Table S2 | 4-9 |
| Table S3 | 10-13 |


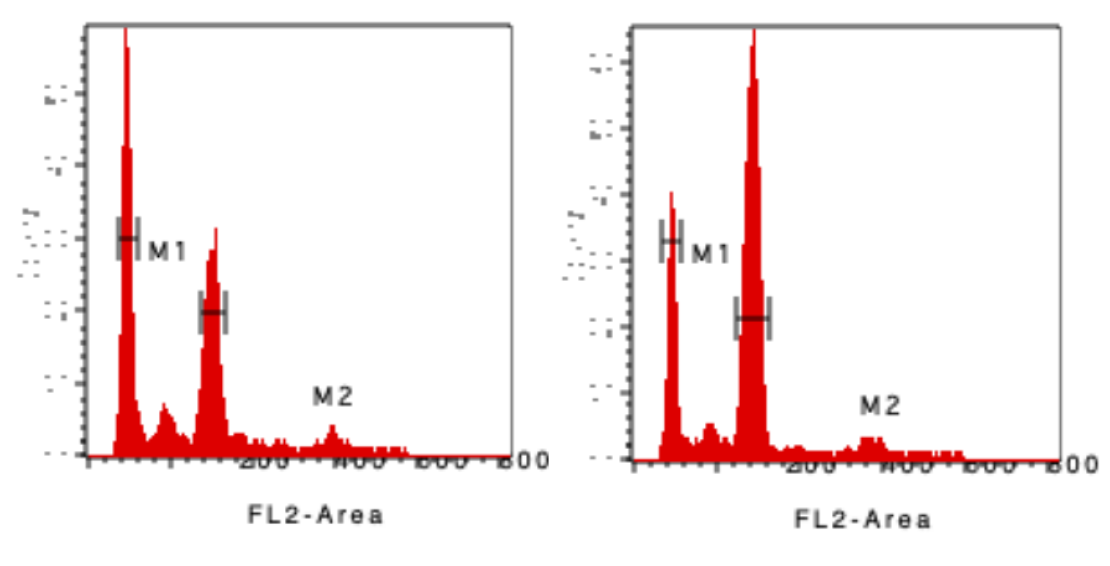


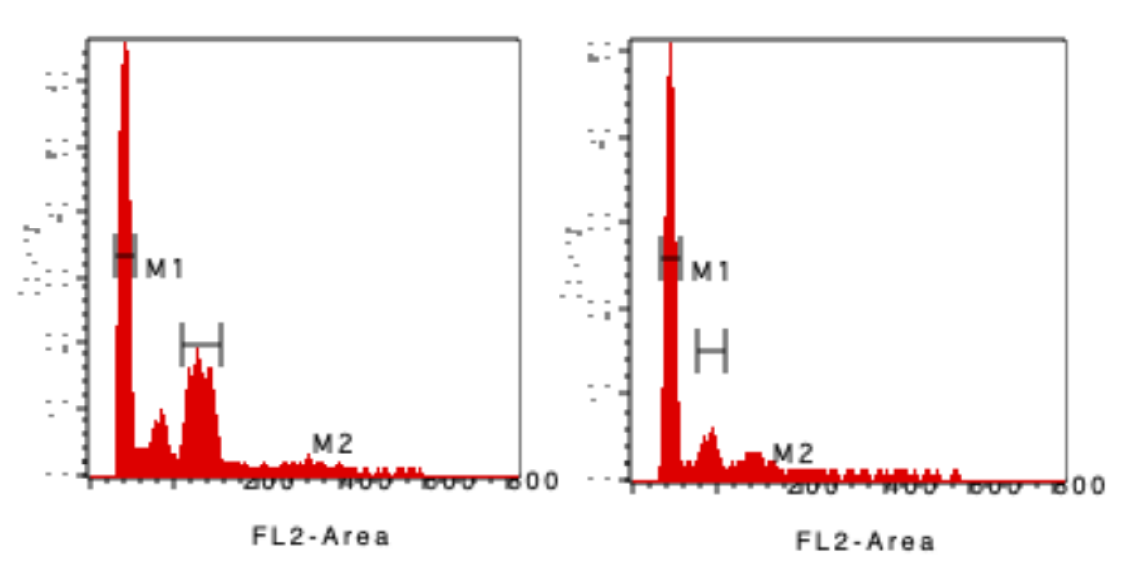


# Fig. S1 Flow cytometry estimation of the genome size. The fruit fly D. melanogaster (a, M2 channel) and the tomato pinworm, *T. absoluta* (b, M2 channel).

# Table S1 source of protein sequence of 22 insects. These accessions were: *T. absoluta* (this study), *Phthorimaea operculella* (GCA_024500475.1, from NCBI), *Pectinophora gossypiella* (GCA_024362695.1, from NCBI), *Antheraea yamamai* (IBG_00066, from InsectBase, http://v2.insect-genome.com/Organism/66), *Manduca sexta* (IBG_00533, from InsectBase), *Bombyx mori* (IBG_00145, from InsectBase), *Helicoverpa armigera* (IBG_00442, from InsectBase), *Spodoptera frugiperda* (IBG_00715, from InsectBase), *Operophtera brumata* (IBG_00590, from InsectBase), *Danaus plexippus* (IBG_00230, from InsectBase), *Papilio xuthus* (IBG_00614, from InsectBase), *Cydia pomonella* (IBG_00224, from InsectBase), *Plutella xylostella* (IBG_00646, from InsectBase), *Stenopsyche tienmushanensis* (IBG_00717, from InsectBase), *Anopheles gambiae* (IBG_00050, from InsectBase), *Drosophila melanogaster* (IBG_00296, from InsectBase), *Tribolium castaneum* (IBG_00768, from InsectBase), *Leptinotarsa decemlineata* (IBG_00487, from InsectBase), *Apis mellifera* (IBG_00083, from InsectBase), *Nasonia vitripennis* (IBG_00564, from InsectBase), *Melanaphis sacchari* (IBG_00547, from InsectBase), and *Rhodnius prolixus* (IBG_00685, from InsectBase).

| Species | Order | Accession |
| --- | --- | --- |
| *Tuta absoluta* | Lepidoptera | / |
| *Phthorimaea operculella* | Lepidoptera | GCA_024500475.1 |
| *Pectinophora gossypiella* | Lepidoptera | GCA_024362695.1 |
| *Antheraea yamamai* | Lepidoptera | IBG_00066 |
| *Manduca sexta* | Lepidoptera | IBG_00533 |
| *Bombyx mori* | Lepidoptera | IBG_00145 |
| *Helicoverpa armigera* | Lepidoptera | IBG_00442 |
| *Spodoptera frugiperda* | Lepidoptera | IBG_00715 |
| *Operophtera brumata* | Lepidoptera | IBG_00590 |
| *Danaus plexippus* | Lepidoptera | IBG_00230 |
| *Papilio xuthus* | Lepidoptera | IBG_00614 |
| *Cydia pomonella* | Lepidoptera | IBG_00224 |
| *Plutella xylostella* | Lepidoptera | IBG_00646 |
| *Stenopsyche tienmushanensis* | Trichoptera | IBG_00717 |
| *Anopheles gambiae* | Diptera | IBG_00050 |
| *Drosophila melanogaster* | Diptera | IBG_00296 |
| *Tribolium castaneum* | Coleoptera | IBG_00768 |
| *Leptinotarsa decemlineata* | Coleoptera | IBG_00487 |
| *Apis mellifera* | Hymenoptera | IBG_00083 |
| *Nasonia vitripennis* | Hymenoptera | IBG_00564 |
| *Melanaphis sacchari* | Hemiptera | IBG_00547 |
| *Rhodnius prolixus* | Hemiptera | IBG_00685 |

# Table S2 GO enrichment of expanded genes in the tomato pinworm (*p-*adjusted<0.05, Hypergeometric test, FDR-adjust)

| **GO ID** | **Description** | **GeneRatio** | **BgRatio** | ***p*value** | ***p*-adjusted** | |  |
| --- | --- | --- | --- | --- | --- | --- | --- |
| **# Biological Process** | |  |  |  |  | |  |
| GO:0110077 | vesicle-mediated intercellular transport | 10/38 | 10/8409 | 9.76E-25 | | 2.58E-22 | |
| GO:0010496 | intercellular transport | 10/38 | 16/8409 | 7.67E-21 | | 1.01E-18 | |
| GO:0042595 | behavioral response to starvation | 10/38 | 30/8409 | 2.76E-17 | | 2.43E-15 | |
| GO:0048168 | regulation of neuronal synaptic plasticity | 10/38 | 63/8409 | 1.06E-13 | | 7.00E-12 | |
| GO:0042594 | response to starvation | 15/38 | 297/8409 | 8.70E-13 | | 4.59E-11 | |
| GO:0051028 | mRNA transport | 10/38 | 120/8409 | 8.10E-11 | | 3.56E-09 | |
| GO:0048167 | regulation of synaptic plasticity | 10/38 | 138/8409 | 3.26E-10 | | 1.23E-08 | |
| GO:0060547 | negative regulation of necrotic cell death | 5/38 | 11/8409 | 6.50E-10 | | 1.72E-08 | |
| GO:1900103 | positive regulation of endoplasmic reticulum unfolded protein response | 5/38 | 11/8409 | 6.50E-10 | | 1.72E-08 | |
| GO:1903897 | regulation of PERK-mediated unfolded protein response | 5/38 | 11/8409 | 6.50E-10 | | 1.72E-08 | |
| GO:1902237 | positive regulation of endoplasmic reticulum stress-induced intrinsic apoptotic signaling pathway | 5/38 | 12/8409 | 1.11E-09 | | 2.55E-08 | |
| GO:0031667 | response to nutrient levels | 15/38 | 492/8409 | 1.16E-09 | | 2.55E-08 | |
| GO:0009991 | response to extracellular stimulus | 15/38 | 499/8409 | 1.41E-09 | | 2.86E-08 | |
| GO:0038034 | signal transduction in absence of ligand | 5/38 | 13/8409 | 1.80E-09 | | 2.97E-08 | |
| GO:0097192 | extrinsic apoptotic signaling pathway in absence of ligand | 5/38 | 13/8409 | 1.80E-09 | | 2.97E-08 | |
| GO:1901030 | positive regulation of mitochondrial outer membrane permeabilization involved in apoptotic signaling pathway | 5/38 | 13/8409 | 1.80E-09 | | 2.97E-08 | |
| GO:0003012 | muscle system process | 10/38 | 181/8409 | 4.67E-09 | | 7.25E-08 | |
| GO:0035234 | ectopic germ cell programmed cell death | 5/38 | 16/8409 | 6.04E-09 | | 8.86E-08 | |
| GO:0050657 | nucleic acid transport | 10/38 | 190/8409 | 7.47E-09 | | 9.86E-08 | |
| GO:0050658 | RNA transport | 10/38 | 190/8409 | 7.47E-09 | | 9.86E-08 | |
| GO:0001836 | release of cytochrome c from mitochondria | 5/38 | 17/8409 | 8.53E-09 | | 1.02E-07 | |
| GO:0051900 | regulation of mitochondrial depolarization | 5/38 | 17/8409 | 8.53E-09 | | 1.02E-07 | |
| GO:0051236 | establishment of RNA localization | 10/38 | 194/8409 | 9.13E-09 | | 1.05E-07 | |
| GO:1901028 | regulation of mitochondrial outer membrane permeabilization involved in apoptotic signaling pathway | 5/38 | 18/8409 | 1.18E-08 | | 1.30E-07 | |
| GO:0010940 | positive regulation of necrotic cell death | 5/38 | 20/8409 | 2.12E-08 | | 2.15E-07 | |
| GO:1900119 | positive regulation of execution phase of apoptosis | 5/38 | 20/8409 | 2.12E-08 | | 2.15E-07 | |
| GO:0007157 | heterophilic cell-cell adhesion via plasma membrane cell adhesion molecules | 6/38 | 41/8409 | 2.26E-08 | | 2.21E-07 | |
| GO:1902235 | regulation of endoplasmic reticulum stress-induced intrinsic apoptotic signaling pathway | 5/38 | 21/8409 | 2.77E-08 | | 2.55E-07 | |
| GO:0015931 | nucleobase-containing compound transport | 10/38 | 218/8409 | 2.80E-08 | | 2.55E-07 | |
| GO:0060561 | apoptotic process involved in morphogenesis | 5/38 | 26/8409 | 8.81E-08 | | 7.75E-07 | |
| GO:0003254 | regulation of membrane depolarization | 5/38 | 27/8409 | 1.08E-07 | | 8.62E-07 | |
| GO:1900101 | regulation of endoplasmic reticulum unfolded protein response | 5/38 | 27/8409 | 1.08E-07 | | 8.62E-07 | |
| GO:1900117 | regulation of execution phase of apoptosis | 5/38 | 27/8409 | 1.08E-07 | | 8.62E-07 | |
| GO:0010939 | regulation of necrotic cell death | 5/38 | 28/8409 | 1.31E-07 | | 1.02E-06 | |
| GO:1905898 | positive regulation of response to endoplasmic reticulum stress | 5/38 | 29/8409 | 1.57E-07 | | 1.17E-06 | |
| GO:0006403 | RNA localization | 10/38 | 262/8409 | 1.59E-07 | | 1.17E-06 | |
| GO:0072332 | intrinsic apoptotic signaling pathway by p53 class mediator | 5/38 | 32/8409 | 2.64E-07 | | 1.89E-06 | |
| GO:0006921 | cellular component disassembly involved in execution phase of apoptosis | 5/38 | 37/8409 | 5.63E-07 | | 3.72E-06 | |
| GO:0046902 | regulation of mitochondrial membrane permeability | 5/38 | 37/8409 | 5.63E-07 | | 3.72E-06 | |
| GO:0051402 | neuron apoptotic process | 5/38 | 37/8409 | 5.63E-07 | | 3.72E-06 | |
| GO:0010823 | negative regulation of mitochondrion organization | 5/38 | 38/8409 | 6.46E-07 | | 4.06E-06 | |
| GO:2001244 | positive regulation of intrinsic apoptotic signaling pathway | 5/38 | 38/8409 | 6.46E-07 | | 4.06E-06 | |
| GO:0097191 | extrinsic apoptotic signaling pathway | 5/38 | 39/8409 | 7.39E-07 | | 4.54E-06 | |
| GO:0050804 | modulation of chemical synaptic transmission | 10/38 | 312/8409 | 8.03E-07 | | 4.82E-06 | |
| GO:0099177 | regulation of trans-synaptic signaling | 10/38 | 316/8409 | 9.02E-07 | | 5.29E-06 | |
| GO:1904035 | regulation of epithelial cell apoptotic process | 5/38 | 41/8409 | 9.55E-07 | | 5.48E-06 | |
| GO:0090559 | regulation of membrane permeability | 5/38 | 42/8409 | 1.08E-06 | | 6.07E-06 | |
| GO:0070997 | neuron death | 5/38 | 43/8409 | 1.22E-06 | | 6.70E-06 | |
| GO:0008630 | intrinsic apoptotic signaling pathway in response to DNA damage | 5/38 | 46/8409 | 1.72E-06 | | 9.26E-06 | |
| GO:0010822 | positive regulation of mitochondrion organization | 5/38 | 47/8409 | 1.92E-06 | | 1.01E-05 | |
| GO:0098742 | cell-cell adhesion via plasma-membrane adhesion molecules | 6/38 | 86/8409 | 2.04E-06 | | 1.05E-05 | |
| GO:0008637 | apoptotic mitochondrial changes | 5/38 | 48/8409 | 2.13E-06 | | 1.06E-05 | |
| GO:0070050 | neuron cellular homeostasis | 5/38 | 48/8409 | 2.13E-06 | | 1.06E-05 | |
| GO:0051881 | regulation of mitochondrial membrane potential | 5/38 | 49/8409 | 2.37E-06 | | 1.16E-05 | |
| GO:0048709 | oligodendrocyte differentiation | 5/38 | 52/8409 | 3.20E-06 | | 1.53E-05 | |
| GO:0016239 | positive regulation of macroautophagy | 5/38 | 53/8409 | 3.52E-06 | | 1.66E-05 | |
| GO:0006919 | activation of cysteine-type endopeptidase activity involved in apoptotic process | 5/38 | 57/8409 | 5.06E-06 | | 2.35E-05 | |
| GO:1902742 | apoptotic process involved in development | 5/38 | 58/8409 | 5.52E-06 | | 2.51E-05 | |
| GO:1905897 | regulation of response to endoplasmic reticulum stress | 5/38 | 59/8409 | 6.02E-06 | | 2.69E-05 | |
| GO:0097194 | execution phase of apoptosis | 5/38 | 61/8409 | 7.10E-06 | | 3.12E-05 | |
| GO:0072331 | signal transduction by p53 class mediator | 5/38 | 78/8409 | 2.38E-05 | | 0.000103153 | |
| GO:0008584 | male gonad development | 5/38 | 83/8409 | 3.22E-05 | | 0.00013731 | |
| GO:0046546 | development of primary male sexual characteristics | 5/38 | 84/8409 | 3.42E-05 | | 0.000143212 | |
| GO:0007006 | mitochondrial membrane organization | 5/38 | 86/8409 | 3.83E-05 | | 0.000153204 | |
| GO:0010508 | positive regulation of autophagy | 5/38 | 86/8409 | 3.83E-05 | | 0.000153204 | |
| GO:0043280 | positive regulation of cysteine-type endopeptidase activity involved in apoptotic process | 5/38 | 86/8409 | 3.83E-05 | | 0.000153204 | |
| GO:0010821 | regulation of mitochondrion organization | 5/38 | 92/8409 | 5.30E-05 | | 0.000208955 | |
| GO:2001056 | positive regulation of cysteine-type endopeptidase activity | 5/38 | 93/8409 | 5.59E-05 | | 0.000216868 | |
| GO:2001235 | positive regulation of apoptotic signaling pathway | 5/38 | 94/8409 | 5.88E-05 | | 0.000224993 | |
| GO:0010623 | programmed cell death involved in cell development | 5/38 | 100/8409 | 7.91E-05 | | 0.000298262 | |
| GO:0097193 | intrinsic apoptotic signaling pathway | 5/38 | 105/8409 | 9.98E-05 | | 0.000365864 | |
| GO:2001242 | regulation of intrinsic apoptotic signaling pathway | 5/38 | 105/8409 | 9.98E-05 | | 0.000365864 | |
| GO:0043524 | negative regulation of neuron apoptotic process | 5/38 | 108/8409 | 0.00011406 | | 0.000412489 | |
| GO:0010950 | positive regulation of endopeptidase activity | 5/38 | 109/8409 | 0.000119152 | | 0.000425082 | |
| GO:0098609 | cell-cell adhesion | 6/38 | 184/8409 | 0.000155956 | | 0.000548964 | |
| GO:0010212 | response to ionizing radiation | 5/38 | 120/8409 | 0.000187483 | | 0.000651258 | |
| GO:0010952 | positive regulation of peptidase activity | 5/38 | 122/8409 | 0.000202589 | | 0.000694591 | |
| GO:0051480 | regulation of cytosolic calcium ion concentration | 5/38 | 133/8409 | 0.00030299 | | 0.001025504 | |
| GO:0016241 | regulation of macroautophagy | 5/38 | 141/8409 | 0.000396981 | | 0.001326619 | |
| GO:2001234 | negative regulation of apoptotic signaling pathway | 5/38 | 142/8409 | 0.000410122 | | 0.001353403 | |
| GO:0043281 | regulation of cysteine-type endopeptidase activity involved in apoptotic process | 5/38 | 143/8409 | 0.000423591 | | 0.001380592 | |
| GO:0010001 | glial cell differentiation | 5/38 | 147/8409 | 0.000480841 | | 0.001548075 | |
| GO:0043523 | regulation of neuron apoptotic process | 5/38 | 158/8409 | 0.000668557 | | 0.002126494 | |
| GO:0006839 | mitochondrial transport | 5/38 | 159/8409 | 0.000688004 | | 0.002162299 | |
| GO:0009267 | cellular response to starvation | 5/38 | 160/8409 | 0.000707874 | | 0.002198573 | |
| GO:1901215 | negative regulation of neuron death | 5/38 | 161/8409 | 0.000728171 | | 0.002235316 | |
| GO:2000116 | regulation of cysteine-type endopeptidase activity | 5/38 | 162/8409 | 0.000748903 | | 0.002272532 | |
| GO:0097190 | apoptotic signaling pathway | 5/38 | 164/8409 | 0.00079169 | | 0.002375071 | |
| GO:0046661 | male sex differentiation | 5/38 | 170/8409 | 0.000931089 | | 0.002761883 | |
| GO:0022411 | cellular component disassembly | 6/38 | 283/8409 | 0.00153434 | | 0.004500731 | |
| **# Cellular Component** | |  |  |  | |  | |
| GO:0098975 | postsynapse of neuromuscular junction | 10/33 | 29/8108 | 5.25E-18 | | 3.93E-16 | |
| GO:1903561 | extracellular vesicle | 12/33 | 77/8108 | 6.67E-17 | | 1.97E-15 | |
| GO:0043230 | extracellular organelle | 12/33 | 78/8108 | 7.87E-17 | | 1.97E-15 | |
| GO:0031594 | neuromuscular junction | 10/33 | 143/8108 | 1.39E-10 | | 2.61E-09 | |
| GO:0030017 | sarcomere | 10/33 | 175/8108 | 1.02E-09 | | 1.53E-08 | |
| GO:0030016 | myofibril | 10/33 | 192/8108 | 2.53E-09 | | 3.16E-08 | |
| GO:0044449 | obsolete contractile fiber part | 10/33 | 198/8108 | 3.41E-09 | | 3.66E-08 | |
| GO:0043292 | contractile fiber | 10/33 | 215/8108 | 7.58E-09 | | 7.10E-08 | |
| GO:0055038 | recycling endosome membrane | 5/33 | 29/8108 | 9.02E-08 | | 7.51E-07 | |
| GO:0098794 | postsynapse | 10/33 | 304/8108 | 2.04E-07 | | 1.53E-06 | |
| GO:0005801 | cis-Golgi network | 5/33 | 37/8108 | 3.23E-07 | | 2.21E-06 | |
| GO:0099081 | supramolecular polymer | 11/33 | 428/8108 | 5.25E-07 | | 3.03E-06 | |
| GO:0099512 | supramolecular fiber | 11/33 | 428/8108 | 5.25E-07 | | 3.03E-06 | |
| GO:0099080 | supramolecular complex | 11/33 | 432/8108 | 5.77E-07 | | 3.09E-06 | |
| GO:0031901 | early endosome membrane | 5/33 | 55/8108 | 2.45E-06 | | 1.23E-05 | |
| GO:0032588 | trans-Golgi network membrane | 5/33 | 61/8108 | 4.12E-06 | | 1.93E-05 | |
| GO:0005741 | mitochondrial outer membrane | 5/33 | 108/8108 | 6.74E-05 | | 0.000297266 | |
| GO:0055037 | recycling endosome | 5/33 | 112/8108 | 8.02E-05 | | 0.000334003 | |
| GO:0031968 | organelle outer membrane | 5/33 | 131/8108 | 0.000168325 | | 0.000664439 | |
| GO:0019867 | outer membrane | 5/33 | 137/8108 | 0.000207657 | | 0.000778714 | |
| GO:0005802 | trans-Golgi network | 5/33 | 154/8108 | 0.000357748 | | 0.00127767 | |
| GO:0010008 | endosome membrane | 5/33 | 196/8108 | 0.001073132 | | 0.003658405 | |
| GO:0005769 | early endosome | 5/33 | 202/8108 | 0.001228176 | | 0.00400492 | |
| GO:0044440 | obsolete endosomal part | 5/33 | 223/8108 | 0.001904136 | | 0.005950426 | |
| GO:0005743 | mitochondrial inner membrane | 6/33 | 357/8108 | 0.002818681 | | 0.008456043 | |
| GO:0019866 | organelle inner membrane | 6/33 | 377/8108 | 0.003697129 | | 0.010664794 | |
| GO:0000139 | Golgi membrane | 5/33 | 293/8108 | 0.006155428 | | 0.01709841 | |
| **# Molecular Function** | |  |  |  | |  | |
| GO:0000981 | DNA-binding transcription factor activity, RNA polymerase II-specific | 13/31 | 465/7396 | 1.47E-08 | | 4.28E-07 | |
| GO:0003729 | mRNA binding | 2023/10/31 | 262/7396 | 6.03E-08 | | 8.74E-07 | |
| GO:0031625 | ubiquitin protein ligase binding | 2023/5/31 | 207/7396 | 0.001535676 | | 0.014282276 | |
| GO:0044389 | ubiquitin-like protein ligase binding | 2023/5/31 | 219/7396 | 0.001969969 | | 0.014282276 | |
| GO:0046982 | protein heterodimerization activity | 2023/5/31 | 298/7396 | 0.007368294 | | 0.042736108 | |
| GO:0003707 | steroid hormone receptor activity | 2023/1/31 | 11/7396 | 0.045181711 | | 0.217136924 | |
| GO:0019904 | protein domain specific binding | 2023/5/31 | 494/7396 | 0.052412361 | | 0.217136924 | |
| GO:0000981 | DNA-binding transcription factor activity, RNA polymerase II-specific | 13/31 | 465/7396 | 1.47E-08 | | 4.28E-07 | |
| GO:0003729 | mRNA binding | 2023/10/31 | 262/7396 | 6.03E-08 | | 8.74E-07 | |
| GO:0031625 | ubiquitin protein ligase binding | 2023/5/31 | 207/7396 | 0.001535676 | | 0.014282276 | |
| GO:0044389 | ubiquitin-like protein ligase binding | 2023/5/31 | 219/7396 | 0.001969969 | | 0.014282276 | |
| GO:0046982 | protein heterodimerization activity | 2023/5/31 | 298/7396 | 0.007368294 | | 0.042736108 | |
| GO:0003707 | steroid hormone receptor activity | 2023/1/31 | 11/7396 | 0.045181711 | | 0.217136924 | |
| GO:0019904 | protein domain specific binding | 2023/5/31 | 494/7396 | 0.052412361 | | 0.217136924 | |

# Table S3 GO enrichment of contracted genes in the tomato pinworm (*p-*adjusted<0.05, Hypergeometric test, FDR-adjust)

| **GO ID** | **Description** | **GeneRatio** | **BgRatio** | ***p*value** | ***p*-adjusted** | |  |
| --- | --- | --- | --- | --- | --- | --- | --- |
| **# Biological Process** | |  |  |  |  | |  |
| GO:0006711 | estrogen catabolic process | 8/24 | 11/8409 | 1.95E-19 | | 2.21E-18 | |
| GO:0006789 | bilirubin conjugation | 8/24 | 11/8409 | 1.95E-19 | | 2.21E-18 | |
| GO:0009698 | phenylpropanoid metabolic process | 8/24 | 11/8409 | 1.95E-19 | | 2.21E-18 | |
| GO:0009804 | coumarin metabolic process | 8/24 | 11/8409 | 1.95E-19 | | 2.21E-18 | |
| GO:0018879 | biphenyl metabolic process | 8/24 | 11/8409 | 1.95E-19 | | 2.21E-18 | |
| GO:0046226 | coumarin catabolic process | 8/24 | 11/8409 | 1.95E-19 | | 2.21E-18 | |
| GO:0046271 | phenylpropanoid catabolic process | 8/24 | 11/8409 | 1.95E-19 | | 2.21E-18 | |
| GO:0051552 | flavone metabolic process | 8/24 | 11/8409 | 1.95E-19 | | 2.21E-18 | |
| GO:0052695 | cellular glucuronidation | 8/24 | 11/8409 | 1.95E-19 | | 2.21E-18 | |
| GO:0052696 | flavonoid glucuronidation | 8/24 | 11/8409 | 1.95E-19 | | 2.21E-18 | |
| GO:0052697 | xenobiotic glucuronidation | 8/24 | 11/8409 | 1.95E-19 | | 2.21E-18 | |
| GO:0070980 | biphenyl catabolic process | 8/24 | 11/8409 | 1.95E-19 | | 2.21E-18 | |
| GO:1904223 | regulation of glucuronosyltransferase activity | 8/24 | 11/8409 | 1.95E-19 | | 2.21E-18 | |
| GO:1904224 | negative regulation of glucuronosyltransferase activity | 8/24 | 11/8409 | 1.95E-19 | | 2.21E-18 | |
| GO:2001029 | regulation of cellular glucuronidation | 8/24 | 11/8409 | 1.95E-19 | | 2.21E-18 | |
| GO:2001030 | negative regulation of cellular glucuronidation | 8/24 | 11/8409 | 1.95E-19 | | 2.21E-18 | |
| GO:0009812 | flavonoid metabolic process | 8/24 | 12/8409 | 5.85E-19 | | 6.23E-18 | |
| GO:0016042 | lipid catabolic process | 15/24 | 224/8409 | 1.60E-18 | | 1.61E-17 | |
| GO:0006787 | porphyrin-containing compound catabolic process | 8/24 | 15/8409 | 7.57E-18 | | 5.96E-17 | |
| GO:0033015 | tetrapyrrole catabolic process | 8/24 | 15/8409 | 7.57E-18 | | 5.96E-17 | |
| GO:0042167 | heme catabolic process | 8/24 | 15/8409 | 7.57E-18 | | 5.96E-17 | |
| GO:0042573 | retinoic acid metabolic process | 8/24 | 15/8409 | 7.57E-18 | | 5.96E-17 | |
| GO:0046149 | pigment catabolic process | 8/24 | 15/8409 | 7.57E-18 | | 5.96E-17 | |
| GO:0006631 | fatty acid metabolic process | 15/24 | 255/8409 | 1.15E-17 | | 8.66E-17 | |
| GO:0042178 | xenobiotic catabolic process | 8/24 | 16/8409 | 1.51E-17 | | 1.05E-16 | |
| GO:0071394 | cellular response to testosterone stimulus | 8/24 | 16/8409 | 1.51E-17 | | 1.05E-16 | |
| GO:0006706 | steroid catabolic process | 8/24 | 17/8409 | 2.85E-17 | | 1.91E-16 | |
| GO:0006063 | uronic acid metabolic process | 8/24 | 18/8409 | 5.12E-17 | | 2.99E-16 | |
| GO:0006953 | acute-phase response | 8/24 | 18/8409 | 5.12E-17 | | 2.99E-16 | |
| GO:0008210 | estrogen metabolic process | 8/24 | 18/8409 | 5.12E-17 | | 2.99E-16 | |
| GO:0019585 | glucuronate metabolic process | 8/24 | 18/8409 | 5.12E-17 | | 2.99E-16 | |
| GO:0071378 | cellular response to growth hormone stimulus | 8/24 | 19/8409 | 8.83E-17 | | 4.99E-16 | |
| GO:0008209 | androgen metabolic process | 8/24 | 20/8409 | 1.47E-16 | | 7.39E-16 | |
| GO:0042537 | benzene-containing compound metabolic process | 8/24 | 20/8409 | 1.47E-16 | | 7.39E-16 | |
| GO:0045922 | negative regulation of fatty acid metabolic process | 8/24 | 20/8409 | 1.47E-16 | | 7.39E-16 | |
| GO:0045939 | negative regulation of steroid metabolic process | 8/24 | 20/8409 | 1.47E-16 | | 7.39E-16 | |
| GO:0034754 | cellular hormone metabolic process | 11/24 | 92/8409 | 3.23E-16 | | 1.58E-15 | |
| GO:0071392 | cellular response to estradiol stimulus | 8/24 | 25/8409 | 1.25E-15 | | 5.96E-15 | |
| GO:0002526 | acute inflammatory response | 8/24 | 26/8409 | 1.80E-15 | | 8.37E-15 | |
| GO:0032787 | monocarboxylic acid metabolic process | 15/24 | 362/8409 | 2.23E-15 | | 1.01E-14 | |
| GO:0071361 | cellular response to ethanol | 8/24 | 28/8409 | 3.58E-15 | | 1.58E-14 | |
| GO:0017001 | antibiotic catabolic process | 8/24 | 29/8409 | 4.93E-15 | | 2.12E-14 | |
| GO:0042447 | hormone catabolic process | 8/24 | 30/8409 | 6.71E-15 | | 2.70E-14 | |
| GO:0051187 | obsolete cofactor catabolic process | 8/24 | 30/8409 | 6.71E-15 | | 2.70E-14 | |
| GO:0060416 | response to growth hormone | 8/24 | 30/8409 | 6.71E-15 | | 2.70E-14 | |
| GO:0010817 | regulation of hormone levels | 15/24 | 392/8409 | 7.31E-15 | | 2.88E-14 | |
| GO:0001523 | retinoid metabolic process | 8/24 | 34/8409 | 2.07E-14 | | 7.96E-14 | |
| GO:0042168 | heme metabolic process | 8/24 | 35/8409 | 2.68E-14 | | 1.01E-13 | |
| GO:0016101 | diterpenoid metabolic process | 8/24 | 36/8409 | 3.43E-14 | | 1.27E-13 | |
| GO:0010677 | negative regulation of cellular carbohydrate metabolic process | 8/24 | 37/8409 | 4.37E-14 | | 1.55E-13 | |
| GO:0033540 | fatty acid beta-oxidation using acyl-CoA oxidase | 7/24 | 20/8409 | 4.46E-14 | | 1.55E-13 | |
| GO:0006805 | xenobiotic metabolic process | 10/24 | 97/8409 | 4.46E-14 | | 1.55E-13 | |
| GO:0006778 | porphyrin-containing compound metabolic process | 8/24 | 38/8409 | 5.53E-14 | | 1.89E-13 | |
| GO:0045912 | negative regulation of carbohydrate metabolic process | 8/24 | 40/8409 | 8.67E-14 | | 2.91E-13 | |
| GO:0042445 | hormone metabolic process | 11/24 | 152/8409 | 9.54E-14 | | 3.14E-13 | |
| GO:0033013 | tetrapyrrole metabolic process | 8/24 | 41/8409 | 1.08E-13 | | 3.48E-13 | |
| GO:0033574 | response to testosterone | 8/24 | 43/8409 | 1.63E-13 | | 5.16E-13 | |
| GO:0008202 | steroid metabolic process | 11/24 | 173/8409 | 4.02E-13 | | 1.26E-12 | |
| GO:0071466 | cellular response to xenobiotic stimulus | 10/24 | 136/8409 | 1.42E-12 | | 4.35E-12 | |
| GO:0007588 | excretion | 8/24 | 56/8409 | 1.56E-12 | | 4.70E-12 | |
| GO:0006721 | terpenoid metabolic process | 8/24 | 57/8409 | 1.81E-12 | | 5.37E-12 | |
| GO:0045833 | negative regulation of lipid metabolic process | 8/24 | 60/8409 | 2.79E-12 | | 8.14E-12 | |
| GO:0062014 | negative regulation of small molecule metabolic process | 8/24 | 62/8409 | 3.67E-12 | | 1.04E-11 | |
| GO:0071385 | cellular response to glucocorticoid stimulus | 8/24 | 62/8409 | 3.67E-12 | | 1.04E-11 | |
| GO:0006636 | unsaturated fatty acid biosynthetic process | 7/24 | 35/8409 | 3.76E-12 | | 1.05E-11 | |
| GO:0019217 | regulation of fatty acid metabolic process | 8/24 | 63/8409 | 4.20E-12 | | 1.15E-11 | |
| GO:0071384 | cellular response to corticosteroid stimulus | 8/24 | 67/8409 | 7.02E-12 | | 1.90E-11 | |
| GO:0031100 | animal organ regeneration | 8/24 | 69/8409 | 8.97E-12 | | 2.39E-11 | |
| GO:0006720 | isoprenoid metabolic process | 8/24 | 76/8409 | 2.00E-11 | | 5.24E-11 | |
| GO:0009593 | detection of chemical stimulus | 8/24 | 79/8409 | 2.75E-11 | | 7.12E-11 | |
| GO:0019218 | regulation of steroid metabolic process | 8/24 | 81/8409 | 3.38E-11 | | 8.62E-11 | |
| GO:0007608 | sensory perception of smell | 8/24 | 82/8409 | 3.74E-11 | | 9.40E-11 | |
| **# Cellular Component** | |  |  |  | |  | |
| GO:0034663 | endoplasmic reticulum chaperone complex | 8/21 | 17/8108 | 1.06E-17 | | 1.90E-16 | |
| GO:0005640 | nuclear outer membrane | 8/21 | 22/8108 | 1.38E-16 | | 1.24E-15 | |
| GO:0070069 | cytochrome complex | 8/21 | 37/8108 | 1.63E-14 | | 9.80E-14 | |
| GO:0005778 | peroxisomal membrane | 7/21 | 51/8108 | 2.76E-11 | | 9.95E-11 | |
| GO:0031903 | microbody membrane | 7/21 | 51/8108 | 2.76E-11 | | 9.95E-11 | |
| GO:0031968 | organelle outer membrane | 8/21 | 131/8108 | 6.39E-10 | | 1.49E-09 | |
| GO:0044438 | obsolete microbody part | 7/21 | 79/8108 | 6.63E-10 | | 1.49E-09 | |
| GO:0044439 | obsolete peroxisomal part | 7/21 | 79/8108 | 6.63E-10 | | 1.49E-09 | |
| GO:0019867 | outer membrane | 8/21 | 137/8108 | 9.15E-10 | | 1.83E-09 | |
| GO:0031965 | nuclear membrane | 8/21 | 178/8108 | 7.35E-09 | | 1.32E-08 | |
| GO:0005777 | peroxisome | 7/21 | 141/8108 | 3.93E-08 | | 5.90E-08 | |
| GO:0042579 | microbody | 7/21 | 141/8108 | 3.93E-08 | | 5.90E-08 | |
| GO:0005635 | nuclear envelope | 8/21 | 315/8108 | 6.21E-07 | | 8.60E-07 | |
| GO:0034774 | secretory granule lumen | 4/21 | 165/8108 | 0.000755152 | | 0.00097091 | |
| GO:0060205 | cytoplasmic vesicle lumen | 4/21 | 169/8108 | 0.000826215 | | 0.000991458 | |
| GO:0031983 | vesicle lumen | 4/21 | 173/8108 | 0.000901904 | | 0.001014641 | |
| **# Molecular Function** | |  |  |  | |  | |
| GO:0015020 | glucuronosyltransferase activity | 10/24 | 32/7396 | 9.08E-19 | | 2.45E-17 | |
| GO:0001972 | retinoic acid binding | 8/24 | 17/7396 | 7.94E-17 | | 1.07E-15 | |
| GO:0005501 | retinoid binding | 8/24 | 22/7396 | 1.03E-15 | | 7.65E-15 | |
| GO:0019166 | trans-2-enoyl-CoA reductase (NADPH) activity | 7/24 | 12/7396 | 1.13E-15 | | 7.65E-15 | |
| GO:0019840 | isoprenoid binding | 8/24 | 26/7396 | 5.02E-15 | | 2.71E-14 | |
| GO:0004857 | enzyme inhibitor activity | 12/24 | 169/7396 | 2.92E-14 | | 1.21E-13 | |
| GO:0008194 | UDP-glycosyltransferase activity | 10/24 | 83/7396 | 3.13E-14 | | 1.21E-13 | |
| GO:0016628 | oxidoreductase activity, acting on the CH-CH group of donors, NAD or NADP as acceptor | 7/24 | 19/7396 | 7.11E-14 | | 2.40E-13 | |
| GO:0005504 | fatty acid binding | 8/24 | 36/7396 | 9.53E-14 | | 2.86E-13 | |
| GO:0033293 | monocarboxylic acid binding | 8/24 | 44/7396 | 5.50E-13 | | 1.48E-12 | |
| GO:0005496 | steroid binding | 8/24 | 46/7396 | 8.06E-13 | | 1.98E-12 | |
| GO:0016758 | hexosyltransferase activity | 10/24 | 130/7396 | 3.16E-12 | | 7.12E-12 | |
| GO:0005080 | protein kinase C binding | 8/24 | 58/7396 | 5.79E-12 | | 1.20E-11 | |
| GO:0016757 | glycosyltransferase activity | 10/24 | 150/7396 | 1.34E-11 | | 2.59E-11 | |
| GO:0016627 | oxidoreductase activity, acting on the CH-CH group of donors | 7/24 | 51/7396 | 1.53E-10 | | 2.76E-10 | |
| GO:0031406 | carboxylic acid binding | 8/24 | 99/7396 | 4.77E-10 | | 8.05E-10 | |
| GO:0043177 | organic acid binding | 8/24 | 102/7396 | 6.08E-10 | | 9.65E-10 | |
| GO:0004867 | serine-type endopeptidase inhibitor activity | 4/24 | 14/7396 | 8.36E-08 | | 1.25E-07 | |
| GO:0004869 | cysteine-type endopeptidase inhibitor activity | 4/24 | 24/7396 | 8.68E-07 | | 1.23E-06 | |
| GO:0046982 | protein heterodimerization activity | 8/24 | 298/7396 | 2.65E-06 | | 3.57E-06 | |
| GO:0008289 | lipid binding | 8/24 | 318/7396 | 4.30E-06 | | 5.53E-06 | |
| GO:0004866 | endopeptidase inhibitor activity | 4/24 | 43/7396 | 9.67E-06 | | 1.19E-05 | |
| GO:0030414 | peptidase inhibitor activity | 4/24 | 45/7396 | 1.16E-05 | | 1.37E-05 | |
| GO:0061135 | endopeptidase regulator activity | 4/24 | 46/7396 | 1.27E-05 | | 1.43E-05 | |
| GO:0008144 | obsolete drug binding | 8/24 | 397/7396 | 2.21E-05 | | 2.38E-05 | |
| GO:0061134 | peptidase regulator activity | 4/24 | 60/7396 | 3.68E-05 | | 3.83E-05 | |
| GO:0019901 | protein kinase binding | 8/24 | 466/7396 | 7.00E-05 | | 7.00E-05 | |
